# Ischemia induces autophagy of endothelial cells and stimulates angiogenic effects in a hindlimb ischemia mouse model

**DOI:** 10.1038/s41419-020-02849-4

**Published:** 2020-08-14

**Authors:** In-Hye Jeong, Woom-Yee Bae, Jae-Sun Choi, Joo-Won Jeong

**Affiliations:** 1 Department of Biomedical Science, Graduate School, Kyung Hee University, Seoul, 02447 Republic of Korea; 2 Department of Anatomy and Neurobiology, College of Medicine, Kyung Hee University, Seoul, 02447 Republic of Korea

**Keywords:** Macroautophagy, Peripheral vascular disease

## Abstract

Although peripheral artery disease (PAD) is a major health problem, there have been limited advances in medical therapies. In PAD patients, angiogenesis is regarded as a promising therapeutic strategy to promote new arterial vessels and improve perfusion of ischemic tissue. Autophagy plays a critical role in catabolic processes for cell survival under normal and stressful conditions and plays fundamental biological roles in various cellular functions. In the present study, we showed that autophagy in endothelial cells is important for the repair and regeneration of damaged tissues. In a hindlimb ischemia mouse model, autophagy was stimulated in endothelial cells of the quadriceps muscle, and adjacent cells proliferated and regenerated. The autophagy pathway was induced under prolonged hypoxia in endothelial cells, and autophagy increased angiogenic activities. Moreover, conditioned media from endothelial cells blocked autophagy and inhibited the proliferation of muscle cells, suggesting that autophagic stimulation in endothelial cells affects the survival of adjacent cells, such as muscle. Collectively, hypoxia/ischemia-induced autophagy angiogenesis, and the damaged tissue surrounded by neo-vessels was regenerated in an ischemia model. Therefore, we strongly suggest that stimulation of autophagy in endothelial cells may be a potent therapeutic strategy in severe vascular diseases, including PAD.

## Introduction

Peripheral arterial disease (PAD) is a general manifestation of atherosclerosis in which obstruction of arterial flow limits blood supply to both upper and lower extremities, most frequently affecting the lower limbs^1,2^. The worldwide prevalence of PAD is ~20% of individuals aged 50 years or older^1,2^. There have been limited advances in medical therapies, which is a huge burden to symptomatic patients with intermittent claudication and critical limb ischemia who have limited treatment options^3,4^. In patients with PAD, revascularization is the preferred therapeutic strategy, and the main strategy in therapeutic angiogenesis is to promote the development of new arterial vessels and improve perfusion of ischemic tissue^5^. The hindlimb ischemia (HLI) model involves acute interruption of arterial supply and has generally been used as a preclinical method to assess angiogenic and arteriogenic regulation in PAD^6,7^.

Angiogenesis, which is the sprouting of new microvessels from the existing vasculature, is an essential physiological process that is often stimulated by hypoxia and is required for wound healing and normal embryogenesis^8^. Angiogenesis is regulated by the coordination of a complicated balance of angiogenic growth factors and inhibitors to induce and sustain the endothelial cell migration and proliferation over a limited time period that is required for tissue repair^9^. In humans, angiogenesis takes place at 3–4 days following ischemic damage, including in stroke patients^10^. Moreover, an increase in cerebral vessel density surrounding the infarcted brain area was observed in animal stroke models^11^. In response to tissue ischemia from hypoperfusion or inadequate blood supply following occlusion of large blood vessels, structural alterations in the existing blood vessels or angiogenesis occur^5^. In the case of PAD patients, therapeutic angiogenesis is a promising strategy and a new treatment paradigm.

Autophagy is a complex cellular process of lysosome-mediated turnover of damaged proteins and dysfunctional organelles^12^. Once autophagy is activated, four sequential steps proceed, initiation, elongation, maturation, and fusion, and each step requires specific regulatory proteins and complexes^13^. Microtubule-associated protein light chain 3 (LC3) is a central protein in autophagy and functions in substrate selection and autophagosome biogenesis, and LC3 is the most widely used marker of autophagosomes^14,15^. As an indicator for autophagic flux, p62 is generally used because it interacts with autophagic substrates and delivers them to autophagosomes for degradation and is itself degraded. Therefore, when autophagy is induced, a decrease in p62 is observed; when autophagy is inhibited, p62 accumulates in the cell^14,15^.

Autophagy occurs at basal levels in most tissues for constitutive turnover of subcellular components; however, it is stimulated by various environmental stresses to recycle nutrients and generate energy for cell survival in unfavorable conditions^16^. Despite the protective roles of autophagy in human physiology and various diseases, the role of autophagy in the vasculature is poorly understood. According to previous reports, autophagy plays protective roles in ischemic injury and oxygen-related stress, such as hypoxia^17,18^. However, the relationship between ischemia-induced autophagy and therapeutic angiogenesis during the regeneration of damaged tissue has not been elucidated. Therefore, in this study, we evaluated the regulatory function of autophagy on endothelial cells that were stimulated by ischemic stress in context of angiogenesis.

## Materials and methods

### Materials

Bafilomycin A1 (Baf), 3-methyladenine (3-MA), chloroquine (CQ), and 5-Bromo-2-deoxyuridine (BrdU) were purchased from Sigma Aldrich (St Louis, MO, USA). Antibodies against p62 and LC3 were obtained from Cell Signaling Technology (Beverly, MA, USA). Antibodies against autophagy-related protein 5 (ATG5), ATG7, dystrophin, beclin1, and lysosome-associated membrane protein 2 (LAMP2) were obtained from Santa Cruz Biotechnology (Dallas, TX, USA). Hypoxia-inducible factor (HIF)-1α, CD31, and BrdU antibodies were purchased from BD Pharmingen (San Diego, CA, USA). Hematoxylin solution and the CYTO-ID kit were purchased from Merck (Darmstadt, Germany) and Enzo Biochem (Farmingdale, NY, USA), respectively.

### Cell culture, hypoxia treatment, and transfection

Human dermal microvascular endothelial cells (HMEC-1) were maintained in MCDB131 medium (Gibco, Santa Clara, CA, USA) containing 10% fetal bovine serum (FBS, Cellgro, VA, USA), 1% penicillin/streptomycin (Cellgro, VA, USA), 10 mM l-glutamate (Gibco, Santa Clara, CA, USA), 10 ng/ml epidermal growth factor (Sigma Aldrich, St Louis, MO, USA), and 1 μg/ml hydrocortisone (Sigma Aldrich, St Louis, MO, USA) in a humidified 5% CO_2_ incubator or in a hypoxic chamber (InvivO2, Baker Ruskinn, Bridgend, UK) that was maintained at 1% O_2_, 5% CO_2_, and 94% N_2_. C2C12 cells were maintained in DMEM medium (WelGene, Gyeongbuk, Korea) supplemented with 10% FBS and 1% penicillin/streptomycin. For differentiation, the cells were treated with 5% horse serum for 6 days. Cells were transfected with the mCherry-EGFP-LC3B expression vector using polyethylenimine reagent (Sigma Aldrich, St Louis, MO, USA).

### Western blotting

The cells were harvested and lysed in lysis buffer (10 mM Tris, 10 mM NaCl, and 0.2% NP-40) supplemented with a protease inhibitor cocktail (Sigma Aldrich, St Louis, MO, USA). The cell extracts were separated by SDS-polyacrylamide gel electrophoresis and western blotting was performed according to a standard protocol.

### CYTO-ID staining

CYTO-ID was used to monitor cell autophagy. The cells were exposed to hypoxia for 4 h after treatment with 25 μM CQ, an autophagy inhibitor, for 4 h. The cells were stained with DAPI (2 μg/ml) and CYTO-ID green detection reagent for 30 min according to the manufacturer’s protocol.

### Tube formation assay

Matrigel (150 μL, BD Pharmingen, San Diego, CA, USA) was polymerized on 24-well plates at 37 °C for 30 min. HMEC-1 cells (1 × 10^5^) were seeded on polymerized Matrigel and incubated with 2 mM 3-MA, 25 μM CQ, or 10 nM Baf under hypoxic conditions. After 16 h, morphological changes were observed, and the mesh number was counted.

### Wound migration assay

The layer of HMEC-1 cells on the bottom of the plate was wounded using a micropipette tip. After washing with PBS, fresh medium containing 1 mM thymidine and with 3-MA, CQ or Baf was added, and incubated under hypoxic conditions for 16 h. The migrated cells are expressed as the percentage of migrated cells compared with that of control cultures under normoxic conditions.

### Rat aortic ring sprouting assay

Aortas were excised from 6-week-old SD rats (Daehan Bio-link, Chungbuk, Korea), and fibroadipose tissue was removed from the excised ring. Rings were sliced at a thickness of 1 mm and were then placed on polymerized Matrigel in each well of a 24-well plate and covered with an additional 50 μL of Matrigel. Average sprouting was measured with ImageJ software (National Institutes of Health, Bethesda, MD, USA) after the plates were photographed.

### BrdU incorporation assay

The cells were seeded in 24-well dishes and incubated with conditioned medium for 24 h. Subsequently, BrdU labeling solution was added to each well at a final concentration of 15 µM for 30 min. The cells were then fixed with 3% paraformaldehyde for 15 min and permeabilized with 0.5% triton-X100 in PBS for 10 min at room temperature. The cells were denatured in 1.2 M HCl for 20 min at 37 °C and incubated with peroxidase-labeled anti-BrdU antibody (1:50) at room temperature for 2 h. Next, the specimens were incubated with fluorescently labeled secondary antibodies for 1 h. The nuclei were counterstained with Hoechst 33342 for 10 min.

### Hindlimb ischemia mouse model and ischemia scoring

All animal experiments were approved by the Committee for Care and Use of Laboratory Animals at the Kyung Hee University (KHUASP(SE)-17–144, 11–24–2017). Six-week-old male BALB/c mice (Daehan Bio-link, Chungbuk, Korea) were anesthetized with an intraperitoneal injection of 30 mg/kg Zoletil (Virbac Korea, Seoul, Korea) and 10 mg/kg Rompun (Bayer Korea, Seoul, Korea), and incisions were made. The subcutaneous adipose tissue was transversely incised by Change-A-tip™ cautery (Bovie Medical Corporation, NY, USA) to reveal the basal femoral artery. The femoral artery and vein were then separated from the nerve and closed with a double knot using a 7–0 silk suture. The retractor was removed, and the incision was closed using a 4–0 silk suture. During surgery, body temperature was monitored by rectal probes and maintained at 37 °C using a temperature-controlled isothermal blanket system (Harvard Apparatus, Holliston, MA, USA). The degree of postoperative ischemic hindlimb necrosis injury, if any, was recorded using a semiquantitative scale as previously reported^19^. We used 4–5 mice per group and all mice were analyzed.

### Indocyanine green imaging

After the mice were anesthetized, hairs from the hindlimb and hips were removed. Each mouse was placed in an 830-nm bandpass filter CCD camera (Vieworks, Gyeonggi-do, Korea), and 1 μg/g (wt) Indocyanine green (ICG; Sigma Aldrich, St Louis, MO, USA) was injected into the tail vein and 760-nm lights were used to illuminate the hindlimbs.

### Immunostaining and immunofluorescent staining

Cells and tissues were fixed, and then the specimens were blocked with 1% BSA and 1.5% FBS in PBS-T for 1 h. Appropriate primary antibodies were incubated for 1 h at room temperature, and a washing step was performed. Next, the specimens were incubated with biotinylated secondary antibodies (Vector Laboratories, Burlingame, CA, USA) or fluorescently labeled secondary antibodies for 1 h. For biotinylation, the tissues were incubated with an Elite ABC Kit (Vector Laboratories, Burlingame, CA, USA), and peroxidase activity was visualized by incubation with 0.02% 3,3′-diaminobenzidine solution (DAB, Sigma Aldrich, St Louis, MO, USA). The nuclei were stained with hematoxylin and Hoechst 33342 for 15 min, respectively.

### Image and statistical analysis

All assays were performed in triplicate, and three independent experiments were performed. ImageJ was used to analyze cell counts or stained areas. Statistical significance was analyzed using Student’s *t* tests, and the results are expressed as the means ± S.D. Differences were considered significant at a *p*-value of <0.05.

## Results

### Regional tissue damage is stimulated by hindlimb ischemia

**Fig. 1:**
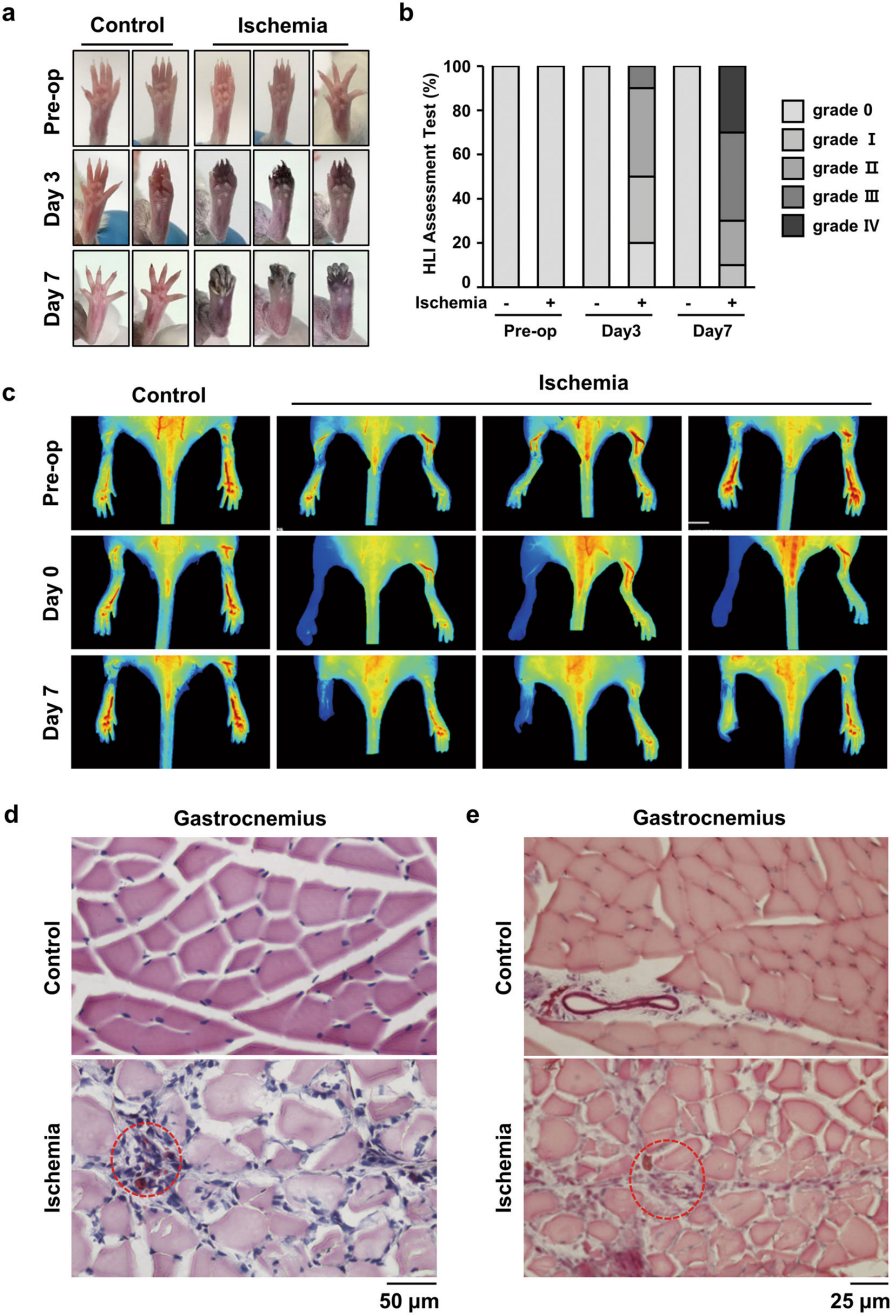
HLI injury induced damage to gastrocnemius tissues in a mouse HLI model. **a** Representative pictures of the foot were taken at days 3 and 7 after HLI injury. **b** The ischemic hindlimb was evaluated using ischemia scoring as described. (grade 0, no damage; grade 1, damaged claws; grade 2, damaged toe; grade 3, damaged to all toes; and grade 4, damaged foot). **c** The blood flow index was examined at the indicated times using ICG. The blood flow index represents the overall blood volume information with respect to time. **d** H&E staining was performed using cross-sectioned gastrocnemius muscle 7 days after injury. The red-dotted circle shows the necrotized region. Scale bar = 50 μm. **e** Gastrocnemius tissue was also stained with Masson, and the red-dotted circle shows fibrosis. Scale bar = 25 μm.

To identify the repair and regeneration mechanism after ischemic injury, we utilized a mouse HLI model. On postoperative days 3 and 7, damage was detected by observing the necrotic feet and toes. Over time, the toes gradually necrotized after ischemic stress (Fig. 1a). The damage after ischemic injury was confirmed by a detailed injury assessment, such as the loss or discoloration of toes (Fig. 1b). Moreover, blood flow was blocked from the tip of the toes to the ankles by ischemic stress (Fig. 1c). To evaluate the histological differences between the control and operation groups, H&E staining was performed using gastrocnemius tissues near the severely damaged feet and toes. We found a large area of muscle necrosis and heterogeneous fiber size in the ischemic group (Fig. 1d). Masson’s trichome staining of the gastrocnemius tissue showed that the area of fibrosis was also increased in the ischemic group (Fig. 1e). Moreover, nuclear translocation and accumulation, which are indicators of muscle damage and fibrosis, were observed by histological detection (Fig. 1d, e). Negative IgG control data for all the immunostaining was presented in Supplementary Fig. 1. These results indicate that ischemic stress blocks peripheral blood flow and stimulates muscle necrosis in a mouse HLI model.

### Tissue regeneration is induced in the quadriceps muscle by ischemic stress

**Fig. 2:**
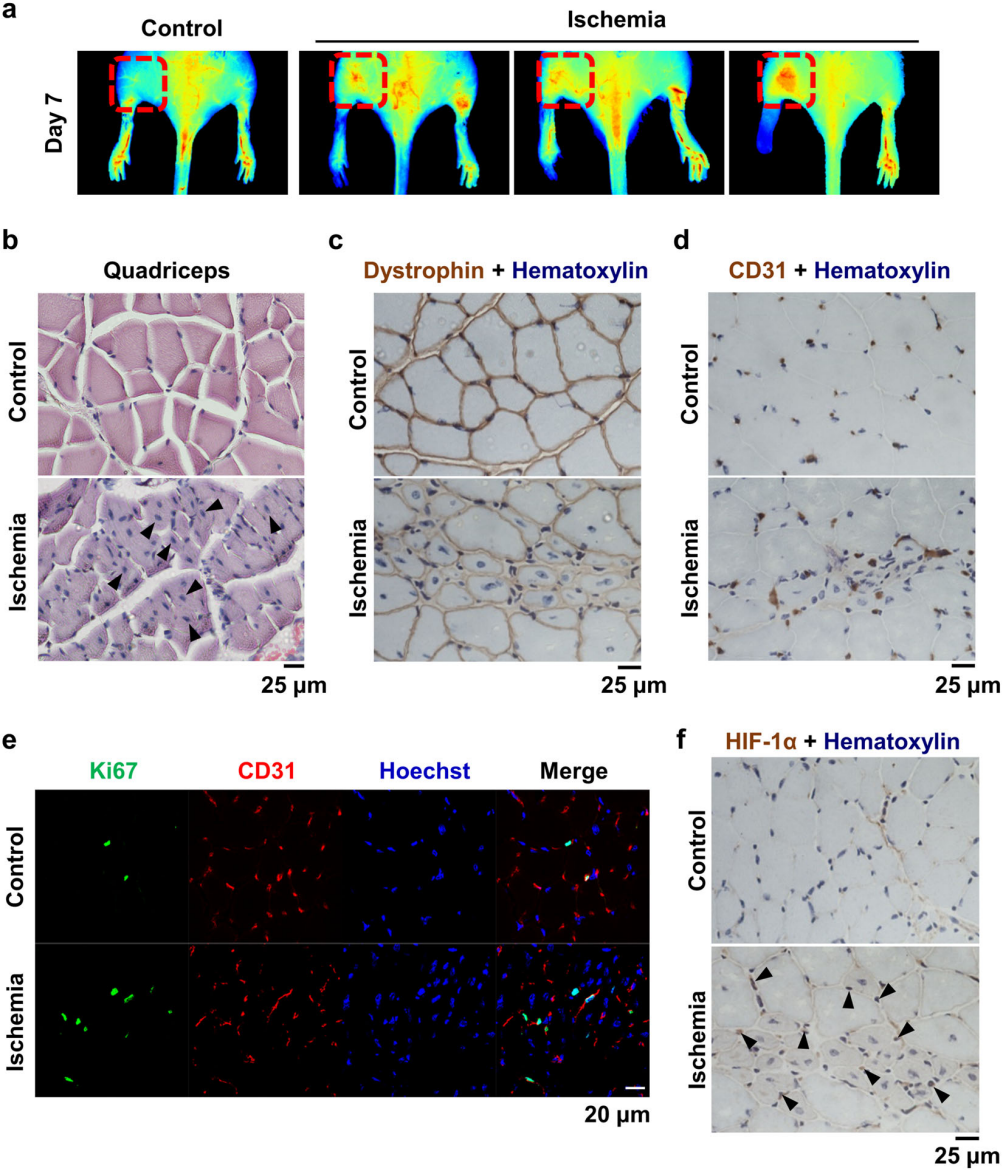
The quadriceps muscle was regenerated in HLI mice. **a** The blood flow index was examined 7 days after surgery, and the upper region was monitored. The red-dotted square shows the blood flow in the quadriceps muscle. **b** Cross-sectioned quadriceps tissue was stained with H&E. Arrow heads indicate centrally shifted nuclei. Scale bar = 25 μm. **c**, **d** Immunostaining of dystrophin and CD31 was performed using quadriceps tissue from HLI mice. Nuclei were counterstained with hematoxylin. Scale bar = 25 μm. **e** Quadriceps tissue was double-fluorescently stained for Ki67 and CD31. Nuclei were counterstained with Hoechst 33342. Scale bar = 20 μm. **f** Immunostaining of HIF-1α was performed, and nuclei were counterstained with hematoxylin. Arrow heads indicate merged signals of HIF-1α and nuclei. Scale bar = 25 μm.

To address whether muscle repair and tissue regeneration occur in an HLI model, we examined the quadriceps tissue that was a distance from the necrotic tissues. On postoperative day 7, blood flow was greatly increased in the area of the quadriceps (Fig. 2a), suggesting that more blood circulation is required to compensate for peripheral blood blockage. When the quadriceps tissue was examined histologically using H&E staining, centrally shifted nuclei, which are indicators of newly proliferating myotubes, were detected in the ischemic group (Fig. 2b). Immunohistochemical analysis of dystrophin and CD31, which are specific markers of muscle cells and endothelial cells, respectively, was performed using quadriceps tissue of ischemic mice. In the ischemic group, the nuclei were located in the center of muscle cells, and endothelial cells were significantly increased compared to those of the control group (Fig. 2c, d). Ki67 is a marker of proliferating cells, and the number of Ki67-positive endothelial cells was increased in the ischemic group (Fig. 2e). HIF-1α was also increased in a similar pattern as that of CD31 expression in the ischemic group (Fig. 2f). Collectively, these results suggest that hypoxia-induced neovascularization stimulates muscle regeneration.

### Endothelial cell autophagy is induced by ischemic conditions

**Fig. 3:**
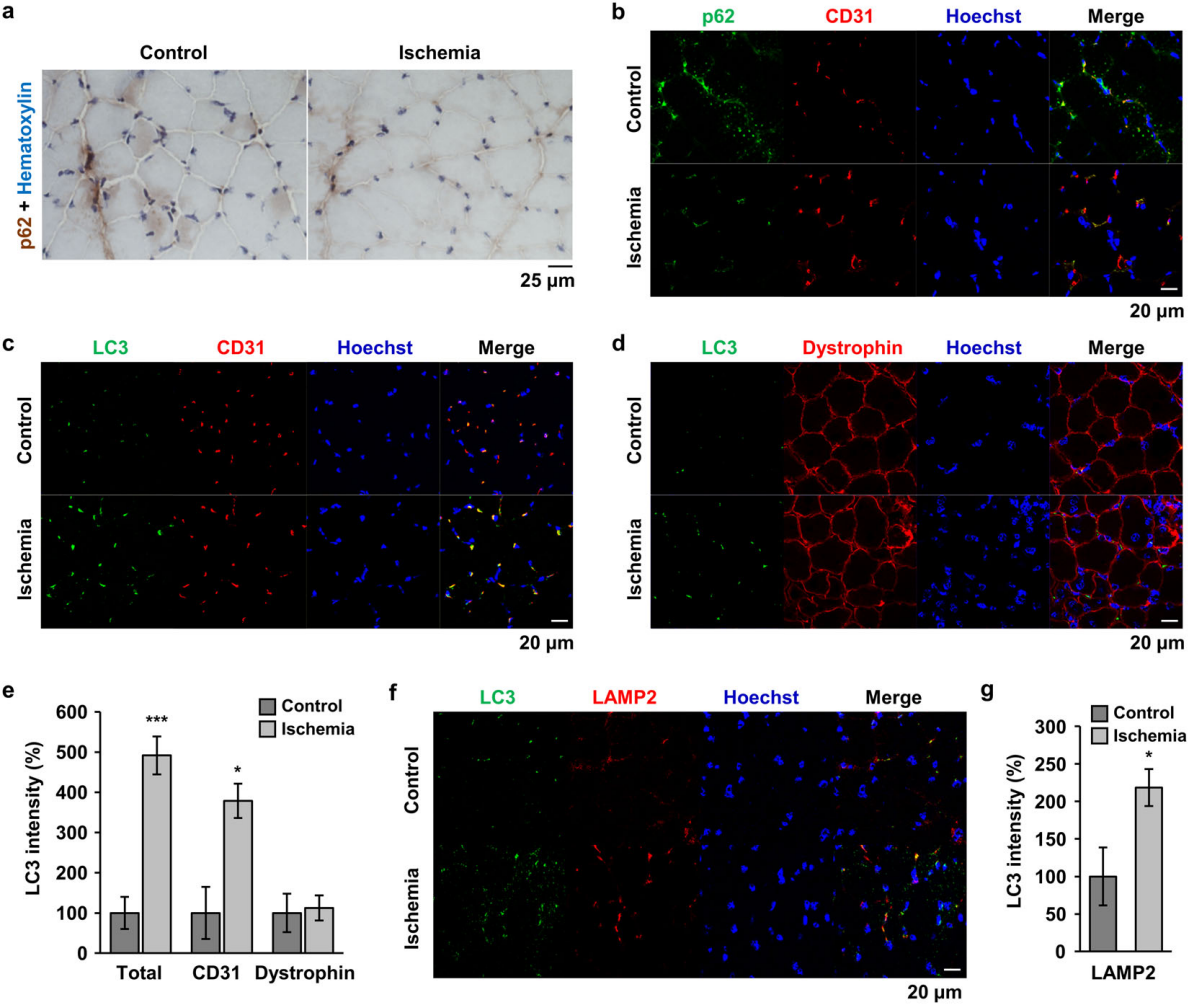
Autophagy was induced in endothelial cells of HLI mice. **a** Immunostaining of p62 was performed using quadriceps tissue from HLI mice. Nuclei were counterstained with hematoxylin. Scale bar = 25 μm. **b**–**d**, **f** Double immunofluorescence staining was performed using specific antibodies as indicated. p62 and CD31 (**b**); LC3 and CD31 (**c**); LC3 and dystrophin (**d**); and LC3 and LAMP2 (**f**). Nuclei were stained with Hoechst 33342. Scale bar = 20 μm. **e**, **g** The total LC3 signal and the overlapped LC3 signal with the indicated protein were quantified using Image J software and plotted. *p < 0.05, ***p < 0.001 versus control.

Autophagy is stimulated by many stresses, such as nutrient deprivation and oxidative injury, in blood vessels to protect surrounding tissues against cell death^20^. Therefore, we examined whether autophagy is induced in quadriceps tissue of HLI mice. Generally, a decrease in p62 is considered an indicator of autophagic flux, and immunohistochemical analysis of p62 was performed. The cytoplasmic expression level of p62 was decreased in the quadriceps of ischemic mice (Fig. 3a), indicating that autophagy was induced under ischemic conditions. However, it was unclear whether autophagic changes occurred in endothelial cells or muscle cells. Therefore, we performed fluorescent double-staining with CD31, and found that signal for p62 overlapped with signals for CD31 and the overlapped p62 signal was decreased under ischemic conditions (Fig. 3b). When LC3 expression was examined by fluorescent double-staining with CD31 or dystrophin, LC3 puncta overlapped CD31 was increased in the tissue of ischemic mice (Fig. 3c, e). In contrast, LC3 signals rarely colocalized with dystrophin expression and the signal overlapped dystrophin was not changed by ischemic injury (Fig. 3d, e), indicating that the stimulation of autophagy under ischemic stress occurred in endothelial cells to preserve the tissue. To prove the activities of autophagy and autophagic flux in this experimental condition, we examined the lysosome-associated LC3 protein by double-staining with LC3 and LAMP2. As shown in Fig. 3f, g, much of LC3 signals were overlapped with LAMP2 signals, and the lysosome-associated LC3 puncta were significantly increased by ischemia.

### Prolonged hypoxia stimulates endothelial cell autophagy

**Fig. 4:**
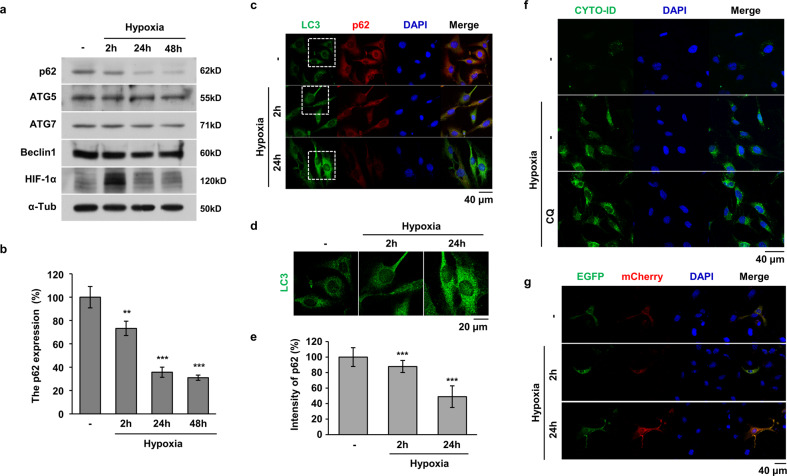
Autophagy was induced by prolonged hypoxia in HMEC-1 cells. **a** HMEC-1 cells were exposed to 1% O_2_ for 2 h, 24 h, and 48 h. Western blotting of whole cell lysates was performed using the indicated antibodies. **b** The relative expression of p62 was quantified and plotted. **P < 0.01 and ***P < 0.001 versus normoxic conditions. **c** HMEC-1 cells were exposed to hypoxia for 2 and 24 h, and LC3 puncta (green) and p62 (red) expression was determined by immunofluorescent staining. Nuclei were counterstained with Hoechst 33342. Scale bar = 40 μm. **d** The white dotted square in **c** was magnified to examine the LC3 puncta. Scale bar = 20 μm. **e** The relative p62 fluorescence intensity of **c** was quantified using ImageJ software. Quantitative values were normalized to 100% normoxic HMEC-1 cells. ***p < 0.001 versus normoxia. **f** HMEC-1 cells were treated with hypoxia and subjected to CYTO-ID (green) staining. Cell nuclei were stained with Hoechst 33342. Scale bar = 40 μm. **g** HMEC-1 cells were transfected with the mCherry-EGFP-LC3B expression vector, and the cells were observed under a fluorescence microscope after hypoxia treatment. Scale bar = 40 μm.

Since ischemia accompanies hypoxia, we investigated whether hypoxia regulates autophagic flux in endothelial cells. After acute or prolonged exposure of HMEC-1 cells to hypoxia, the expression of autophagy-related proteins was examined by western blot analysis. The total protein level of p62 was strongly decreased under prolonged hypoxic exposure for 24 and 48 h; however, a mild change was observed under acute exposure for 2 h (Fig. 4a). The p62 protein level was decreased to ~30% by prolonged hypoxia (Fig. 4b). In contrast to p62, the expression of ATG5, ATG7, and Beclin1 was not changed by hypoxic exposure (Fig. 4a). Since the conversion of LC3 I to LC3 II and formation of LC3 puncta are key steps in the initiation of autophagosome formation^21,22^, LC3 puncta was examined using immunofluorescence staining. A significant increase in LC3 puncta was detected in hypoxia-treated HMEC-1 cells at 24 h but not 2 h (Fig. 4c, d). In the same cells, we also found that p62 expression was decreased (Fig. 4c, e), suggesting that autophagy is stimulated in endothelial cells in response to long-term hypoxic exposure. To examine and monitor autophagic flux, we stained autophagic vacuoles using a CYTO-ID kit and transfected the mCherry-EGFP-LC3 expression vector into HMEC-1 cells. As shown in Fig. 4f, the number of bright green fluorescence-stained vacuoles was increased by hypoxia in the same pattern as that of CQ treatment, which is an inhibitor of autophagy that blocks autophagosome-lysosome fusion. The basis of detection for autophagic flux using the mCherry-EGFP-LC3 expression vector is the increased sensitivity of EGFP to the acidic environment of the autolysosome relative to that of mCherry; namely, cells with higher flux are less green due to fusion of autophagosomes with lysosomes, and the mCherry/EGFP ratio is increased in the cell. The mCherry signal was relatively increased in hypoxia-exposed HMEC-1 cells at 24 h but not at 2 h (Fig. 4g), suggesting that prolonged but not acute hypoxia stimulates autophagic flux in endothelial cells.

### Autophagy increases the angiogenic activities of endothelial cells under hypoxia

**Fig. 5:**
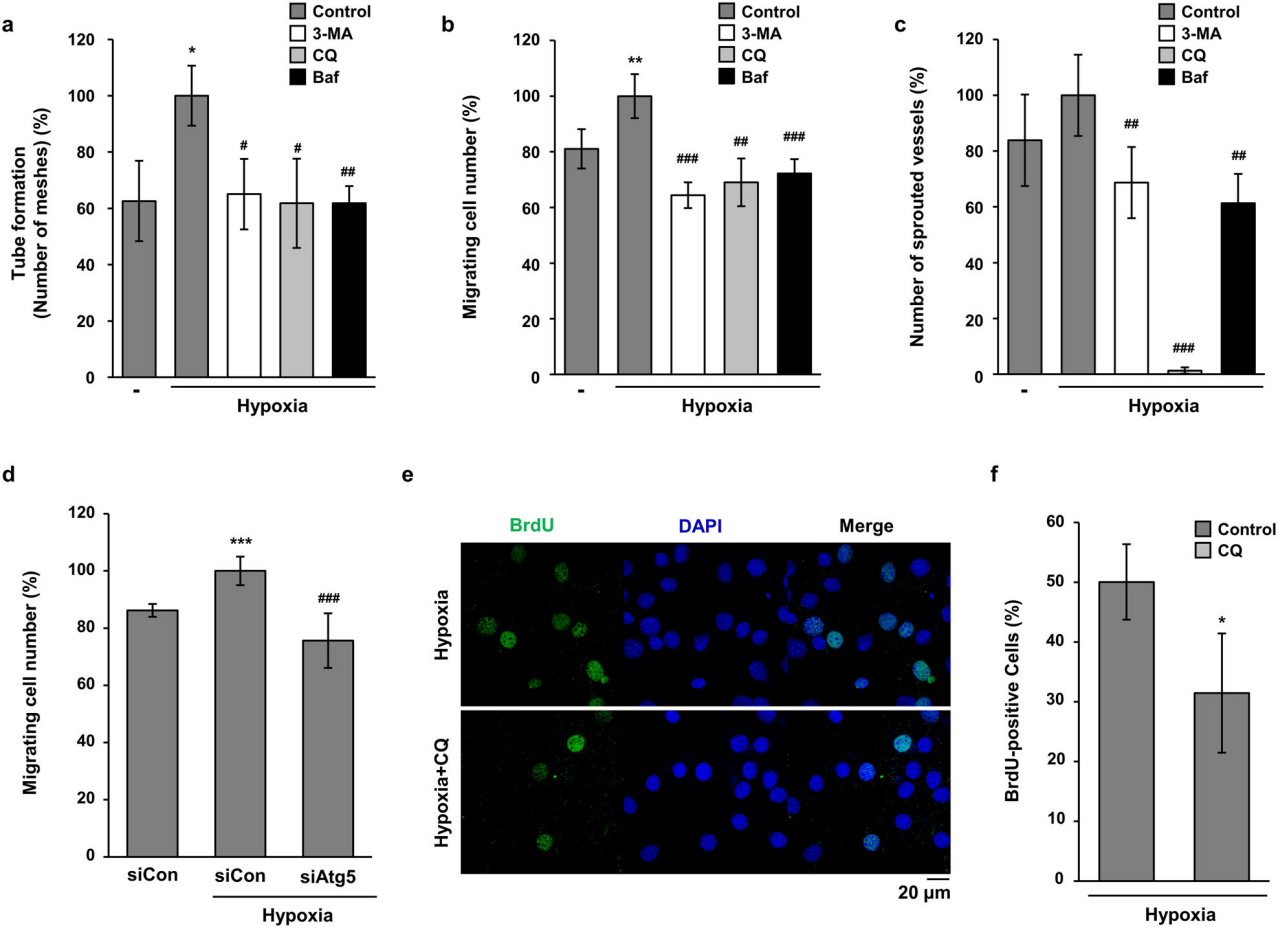
Autophagy inhibitors decreased the angiogenic activities of endothelial cells under hypoxia. **a** HMEC-1 cells were incubated on Matrigel under hypoxia with 3-MA (2 mM), CQ (25 μM), or Baf-A1 (10 nM) for 24 h. The tube formation assay was quantified by counting the meshes from three independent experiments using ImageJ software. *p < 0.05 versus normoxia; ^#^*p* < 0.05 and ^##^*p* < 0.01 versus the hypoxia vehicle. **b** To compare the motility of HMEC-1 cells, a wound migration assay was performed under hypoxia with autophagy inhibitors as indicated. The number of migrated cells from the reference line was counted, quantified, and plotted. **p < 0.01 versus normoxia; ^##^*p* < 0.01 and ^###^*p* < 0.001 versus the hypoxia vehicle. **c** Rat aortic rings were incubated on Matrigel under hypoxia with autophagy inhibitors as indicated. Sprouted microvessels from the aorta were quantified and plotted. ^##^*p* < 0.01 and ^###^*p* < 0.001 versus the hypoxia vehicle. **d** HMEC-1 cells were transfected with siControl (siCon) or siAtg5, and then a wound migration assay was performed under hypoxia. **e** The conditioned media were collected from hypoxia and CQ-treated HMEC-1 cells. C2C12 cells were treated with the conditioned media for 24 h. Proliferative properties of C2C12 cells were evaluated by BrdU incorporation assay and nuclei were stained with Hoechst 33342. Scale bar = 20 μm. **f** The data are expressed as means ± S.D. for three determinations in three independent experiments. *p < 0.05.

To determine the function of hypoxia-induced autophagy in endothelial cells, angiogenesis assays were performed by treatment with autophagy inhibitors. As shown in Fig. 5a and Supplementary Fig. 2a, the hypoxia-induced tube-forming activities of HMEC-1 cells were significantly inhibited by treatment with all tested autophagy inhibitors. Because the migration of endothelial cells is an important step in angiogenesis, we next investigated the effect of autophagy inhibitors on the migratory properties of HMEC-1 cells. As shown in Fig. 5b and Supplementary Fig. 2b, the hypoxia-induced increase in motility was significantly decreased by treatment with 3-MA, CQ, and Baf. Based on the results of the in vitro angiogenesis assays, an ex vivo assay was performed to confirm the regulatory effects of autophagy inhibitors on hypoxia-induced angiogenesis. The results from aortic ring sprouting assay indicated that autophagy inhibitors significantly decreased hypoxia-induced microvessel sprouting, especially CQ, which almost completely blocked sprouting from the aortic ring (Fig. 5c and Supplementary Fig. 2c). To confirm the effects of autophagy inhibitors, we induced Atg5 knockdown in HMEC-1 cells by transfection with siRNA for ATG5 (Supplementary Fig. 3). As shown in Fig. 5d, hypoxia-induced increase in motility of HMEC-1 cells was significantly decreased by transfection with Atg5 siRNA. Because regulation of angiogenesis can affect nearby tissue regeneration, we performed BrdU incorporation assay using C2C12 cells and conditioned media from HMEC-1 cells. As shown in Fig. 5e, the BrdU-positive C2C12 cells were reduced by treatment with CM from CQ-treated HMEC-1 cells under hypoxia. From these data, we assumed that hypoxia-induced autophagy stimulates angiogenesis and consequently helps regeneration of surrounding tissues.

## Discussion

Although angiogenesis is regarded as a promising treatment strategy for patients with PAD and severe vascular diseases, the use of these strategies in clinical application has not been successful so far^5^. In PAD models, the degree of ischemia depends on the location and duration of the artery occlusion^23^. In this study, we used an HLI mouse model to mimic PAD, which resulted in deep distal ischemia. Symptoms of ischemia, such as necrosis of the toes and lime paralysis, were detected in our HLI model. The distal region from the occluded artery, the gastrocnemius, showed severe muscle damage and fibrosis (Fig. 1). However, newly synthesized muscle cells and endothelial cells were observed in the quadriceps, which is the region away from the damage (Fig. 2). Even if the necrotized tissues cannot be restored, more active tissue regeneration and a support system are needed to maintain and compensate for the remaining tissues and blood circulation. Similarly, in the brain tissue of stroke patients, angiogenesis can be detected at 3–4 days following ischemic insults^10^, and an increase in cerebral vessel density surrounding the infarcted brain area was observed in stroke models 3 days after ischemic insults^11,24^. Moreover, patients with increased cerebral blood vessel density show better survival and recovery than those with reduced blood vessel density^9,10,25^. We also found that blood flow was increased in the upper region of the upper limb when blood flow was blocked in the HLI model (Figs. 1, 2). In addition, newly synthesized endothelial cells were increased, and the proliferation of adjacent endothelial cells was increased under these conditions (Fig. 2). These results indicate that angiogenesis is essential for tissue repair and regeneration in tissues that are damage by ischemia or hypoxia.

Autophagy occurs at a basal level to destroy long-lived proteins and organelles under normal physiological conditions and is important for maintaining cellular homeostasis^15^. Autophagy is stimulated by stressful conditions, such as hypoxia and starvation, and protects the cell by degrading cytoplasmic materials to generate amino acids and fatty acids that are used to produce ATP and promote cell survival^26^. Dysregulation of autophagy is correlated with diverse pathologies, such as neurodegeneration, cancer, infection, and vascular diseases, including myocardial ischemia and atherosclerosis^27^. Therefore, autophagy is generally regarded as an essential process for maintaining health and well-being. However, overactivation of autophagy is not always protective and may induce cell death, called autophagic cell death^28,29^. In the PAD model, autophagy was induced in endothelial cells, and prolonged hypoxia also stimulated autophagic induction of endothelial cells (Figs. 3, 4). However, acute hypoxic stress did not regulate the protein levels of autophagy-regulated genes in endothelial cells (Fig. 4). Diseases such as PAD also result in local hypoxia; if this hypoxic stress is continued for a long time, the tissues become necrotized. To survive and regenerate damaged tissues under hypoxia, autophagy is first induced in endothelial cells and may help their surrounding tissues to regenerate. The role of autophagy in endothelial cells has been explored in more detail only in recent years; therefore, the regulatory function and mechanisms of autophagy in endothelial cells have not yet been clarified.

Angiogenesis is a crucial phenomenon that maintains blood nourishment under normal conditions and also participates in the regeneration of ischemic cardiac tissue or impaired peripheral vasculature^30,31^. Recent studies suggest that autophagy serves as a dynamic mechanism that enables endothelial cells to regulate their biogenetic and biosynthetic needs in response to the environment, the presence of angiogenic cues, or injuries^32^. In contrast, deregulated autophagy under stress conditions, such as prolonged nutrient deprivation, may be detrimental to endothelial cells and can lead to autophagic cell death^33–35^. However, the distinct and exact role of autophagy in the modulation of endothelial cells is still controversial and likely dependent on the type of metabolic stress^32^. Under prolonged hypoxic conditions such as PAD, angiogenesis is essential to repair and maintain tissues surrounding severely damaged tissues. In this study, the induction of angiogenesis by hypoxia was decreased by autophagy inhibitors (Fig. 5), suggesting that autophagy stimulates the angiogenic activities of endothelial cells. As mentioned above, dual effects of autophagy on angiogenesis have been reported. Magnolol, an autophagy inducer, inhibits the angiogenic activities of endothelial cells by blocking the Wnt/b-catenin axis^36^. In contrast, starvation induces angiogenic responses by activating VEGF and Akt proteins on endothelial cells^36^. Moreover, AGGF-1, a potent therapeutic candidate for coronary artery disease, induces angiogenesis by promoting autophagy in endothelial cells^36^. We suggest that continued stresses such as prolonged hypoxia and starvation induce autophagy in endothelial cells, which stimulates angiogenesis. Consequently, tissue near the newly synthesized blood vessels is preferentially regenerated by autophagy-induced angiogenesis.

In conclusion, the peripheral damage region, including the gastrocnemius, was necrotized and was not regenerated in HLI mice. However, adjacent remaining tissues, such as the quadriceps, showed more active blood flow, and regeneration occurred. In these regions, autophagy was stimulated, especially in endothelial cells, and autophagic flux was stimulated in endothelial cells by prolonged hypoxia. Moreover, the hypoxia-induced angiogenic activities of endothelial cells were reduced by autophagy inhibitors, indicating that autophagy increases angiogenesis under hypoxic conditions. The results of the present study help to elucidate the important role of autophagy under hypoxic and ischemic conditions and to direct the development of a novel strategy to treat patients with PAD. Moreover, we strongly support the future use of autophagy regulators for the treatment of PAD and related complications.

## Supplementary information

Supplementary Figure 1

Supplementary Figure 2

Supplementary Figure 3

Supplementary Figure Legends
