# Supplementary figures and images for: Ischemia induces autophagy of endothelial cells and stimulates angiogenic effects in a hindlimb ischemia mouse model
Source: Cell Death Dis. 2020 Aug 14;11(8):624. doi: 10.1038/s41419-020-02849-4 (PMC7429831; doi:10.1038/s41419-020-02849-4)

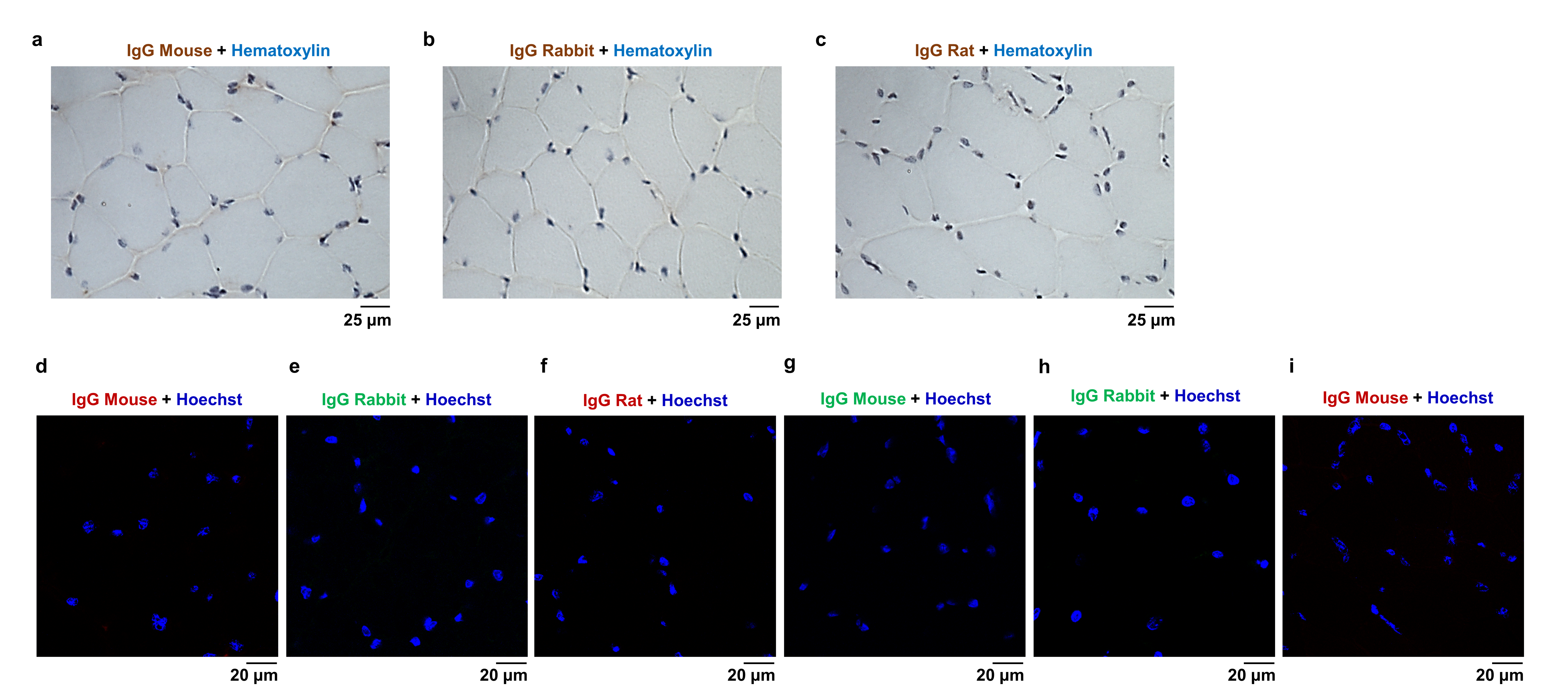

Supplement: Supplementary file 1 — Supplementary Figure 1 [file 41419_2020_2849_MOESM1_ESM.tif]

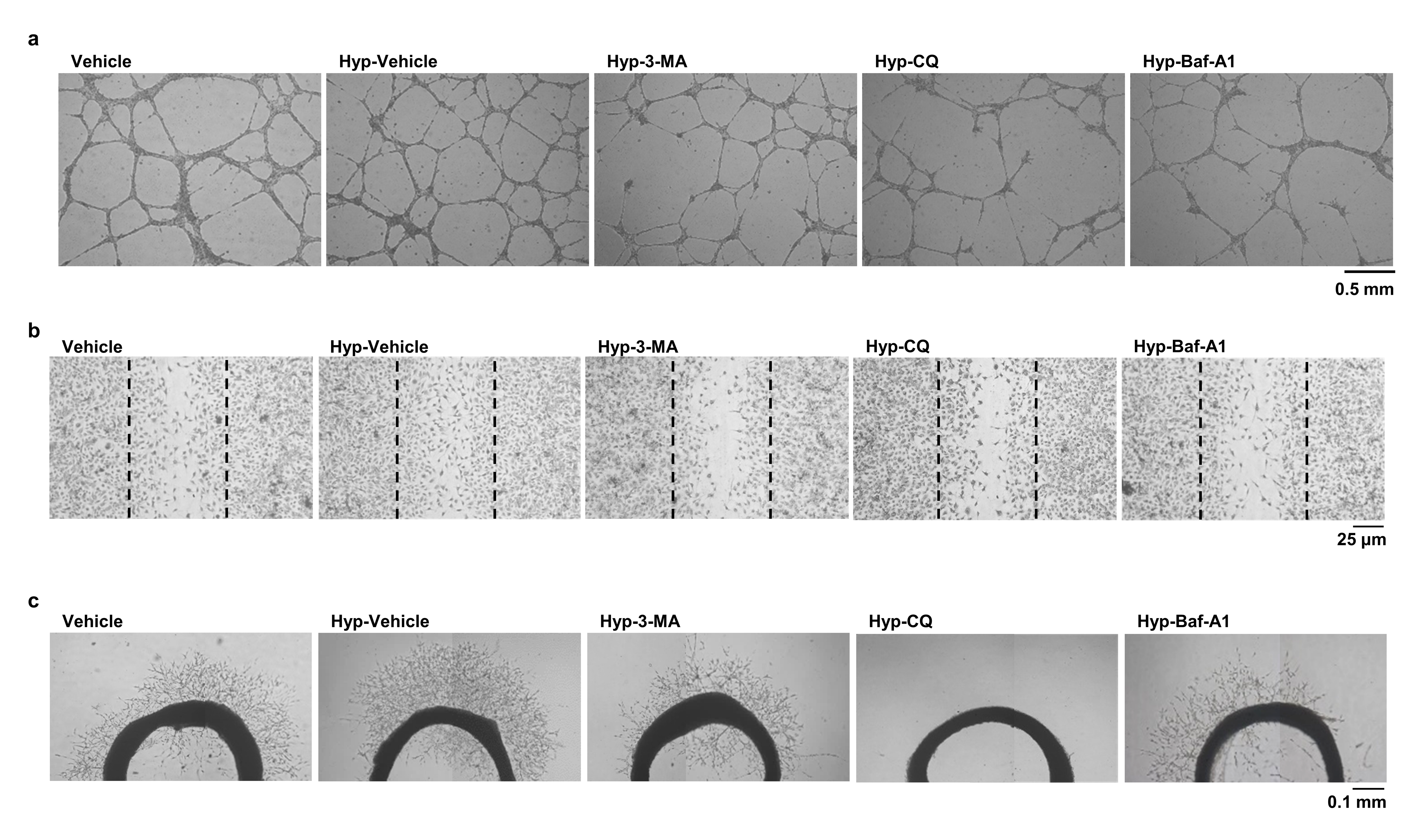

Supplement: Supplementary file 2 — Supplementary Figure 2 [file 41419_2020_2849_MOESM2_ESM.tif]

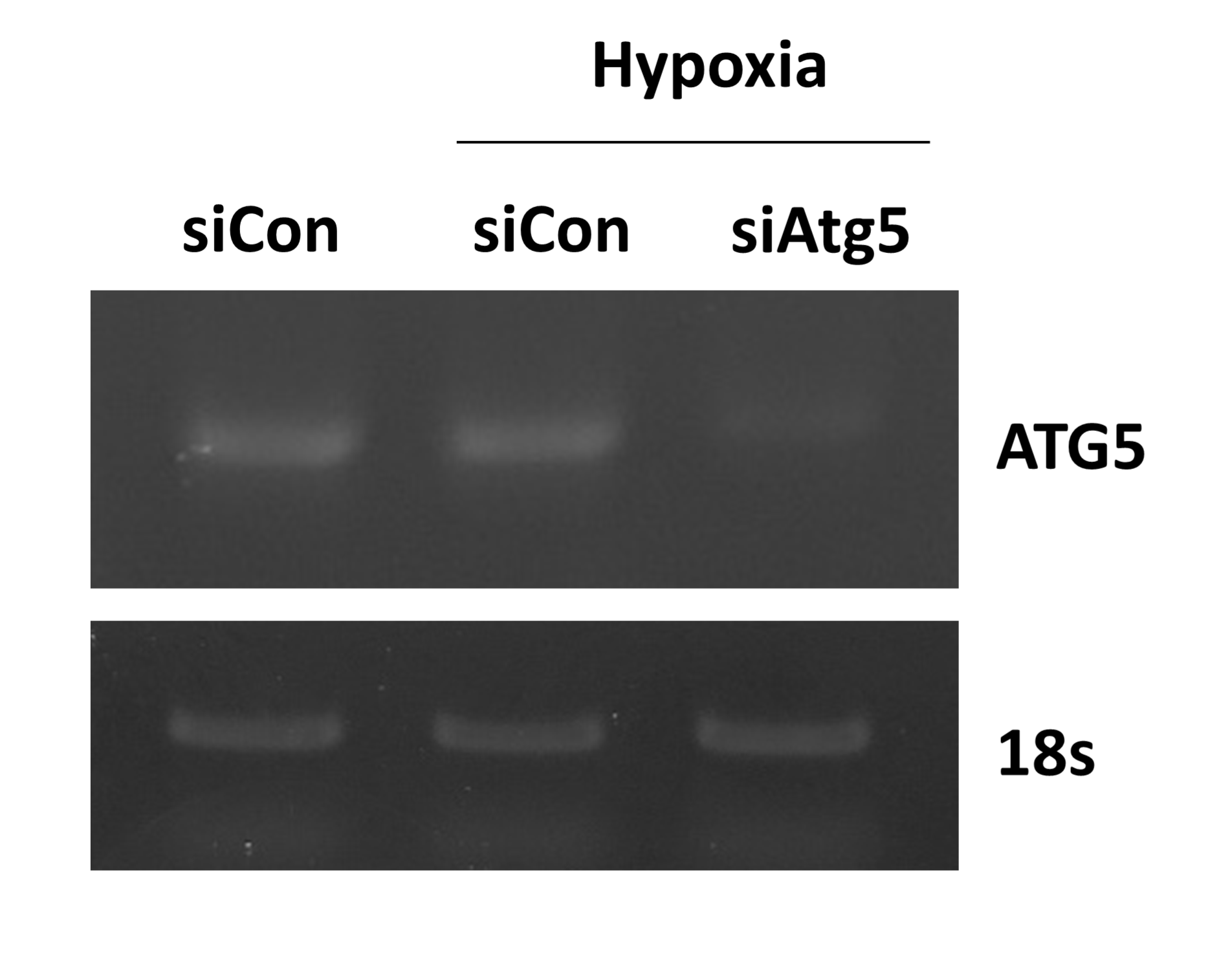

Supplement: Supplementary file 3 — Supplementary Figure 3 [file 41419_2020_2849_MOESM3_ESM.tif]
