# Supplementary material for: Ischemia induces autophagy of endothelial cells and stimulates angiogenic effects in a hindlimb ischemia mouse model
Source: Cell Death Dis. 2020 Aug 14;11(8):624. doi: 10.1038/s41419-020-02849-4 (PMC7429831; doi:10.1038/s41419-020-02849-4)
Supplement: Supplementary file 4 — Supplementary Figure Legends [file 41419_2020_2849_MOESM4_ESM.docx]

Supplementary Figure Legends

**Supplementary Figure 1**

Immunostaining and immunofluorescent staining for each negative IgG control was performed using quadriceps tissues and nuclei were counter stained with hematoxylin or Hoechst 33342. Scale bar = 20 μm. Normal mouse IgG control for Dystrophin immunostaining (a); Normal rabbit IgG control for HIF-1a immunostaining (b); Normal rat IgG control for CD31 immunostaining (c); Normal mouse IgG control for Dystrophin immunofluorescent staining (d); Normal rabbit IgG control for LC3 immunofluorescent staining (e); Normal rat IgG control for CD31 immunofluorescent staining (f); Normal mouse IgG control for p62 immunofluorescent staining (g); Normal rabbit IgG control for Ki67 immunofluorescent staining (h); Normal mouse IgG control for LAMP2 immunofluorescent staining (i)

**Supplementary Figure 2**

(a) HMEC-1 cells were incubated on Matrigel under hypoxia with 3-MA (2 mM), CQ (25 μM) or Baf-A1 (10 nM) for 24 h. Scale bar = 0.5 mm. (b) To compare the motility of HMEC-1 cells, a wound migration assay was performed under hypoxia with autophagy inhibitors as indicated. Scale bar = 25 μm. (c) Rat aortic rings were incubated on Matrigel under hypoxia with autophagy inhibitors as indicated. Scale bar = 1 mm.

**Supplementary Figure 3**

HMEC-1 cells were transfected with siControl (siCon) or siAtg5. Total RNA was isolated from transfected HMEC-1 cells and RT-PCR to determine the mRNA level of Atg5 was performed.
